# Exploration of vitamin D hydroxy metabolites C3 epimers in patients with cardiovascular disease: an observational study

**DOI:** 10.1042/BSR20241558

**Published:** 2025-01-27

**Authors:** Mohamed Abouzid, Łukasz Kruszyna, Julia Kerner, Leonid Kagan, Aniceta Mikulska-Sauermann, Dorota Filipowicz, Matylda Resztak, Franciszek Krzysztof Główka, Marta Karaźniewicz-Łada

**Affiliations:** 1 Department of Physical Pharmacy and Pharmacokinetics, Faculty of Pharmacy, Poznan University of Medical Sciences, 3 Rokietnicka Street, 60-806 Poznan, Poland; 2 Doctoral School, Poznan University of Medical Sciences, Bukowska 70 Street, 60-812 Poznan, Poland; 3 Department of Vascular and Endovascular Surgery, Angiology and Phlebology, Poznan University of Medical Sciences, Długa ½ Street, 60-848 Poznan, Poland; 4 Department of Pharmaceutics and Center of Excellence for Pharmaceutical Translational Research and Education, Ernest Mario School of Pharmacy, Rutgers, The State University of New Jersey, Piscataway, NJ, U.S.A.; 5 Department of Endocrinology, Metabolism and Internal Medicine, Poznan University of Medical Sciences, 49 Przybyszewskiego Street, 60-355 Poznan, Poland

**Keywords:** calcidiol, calcitriol, CVD, epi-hydroxyvitamin D, epimers

## Abstract

Roughly 90% of the Polish population experiences vitamin D deficiency. The 3-epi-25(OH)D_2_ and 3-epi-25(OH)D_3_ are stereoisomers of 25(OH)D_2_ and 25(OH)D_3_, and they can inadvertently be included in measurements of 25(OH)D levels, potentially leading to its overestimating. We aimed to measure 25(OH)D_2_ and 25(OH)D_3_, their epimers 3-epi-25(OH)D_2_ and 3-epi-25(OH)D_3_, and biologically active 1,25(OH)_2_D_3_ in patients with cardiovascular disease and healthy volunteers. We enrolled 27 adult patients with cardiovascular disease (64 ± 15 years) and 35 healthy volunteers (36.37 ± 12.29 years). We used a validated ultra-performance liquid chromatography-mass spectrometry/mass spectrometry (UPLC-MS/MS) method to measure 25(OH)D_2_/_3_ concentrations and their epimers. Plasma concentrations of 1α,25(OH)_2_D_3_ were determined by sensitive and quantitative enzyme immunoassay following intra- and inter-day validation. Vitamin D insufficiency was observed in approximately 52% of the patients and 37% of healthy volunteers. Comparable levels of 25(OH)D_3_ and 25(OH)D_2_ were seen in both groups. The observed levels of the epimeric form 3-epi-25(OH)D_3_ appeared approximately 1.7 times higher in healthy volunteers, accounting for 9% misclassified according to vitamin D status. Also, patients had lower concentrations of 1,25(OH)_2_D_3_, and their 3-epi-25(OH)D_2_ levels were below the detection limit (2 ng/mL). In all studied subjects, 25(OH)D_3_ was negatively correlated with % 3-epi-25(OH)D_3_ (*R*=-0.758; *P*<0.001), and 3-epi-25(OH)D_2_ was negatively correlated with % 3-epi-25(OH)D_2_ (*R* = -0.842; *P* = 0.002). While the mechanism of how vitamin D epimeric forms influence diseases remains unclear, we recommend maintaining 25(OH)D_3_ levels above 20 ng/mL.

## Introduction

Recent research has increasingly focused on vitamin D deficiency [1], recognizing it as a widespread issue often termed a ‘pandemic.’ This deficiency affects approximately 33% of Americans [2], 60% of Canadians [3], and 40% of Europeans [4]. In 2016, a comprehensive prevalence study conducted by Płudowski et al., involving 5755 adults, revealed an alarming statistic: 90% of the Polish population have vitamin D deficiency [5].

Vitamin D deficiency has been implicated in the pathogenesis of acute coronary syndrome [6] and is associated with higher cardiovascular morbidity and mortality rates [7]. Brown et al. have identified several established risk factors for coronary artery disease, and the ongoing discussion revolves around its potential association with vitamin D deficiency [8].

This deficiency is commonly observed among patients with conditions such as myocardial infarction, coronary heart failure, ischemic heart disease, transient ischemic attack, and atrial fibrillation [9]. A recent cohort study involving 10,974 participants highlighted the effectiveness of supplemental vitamin D in reducing in-hospital mortality rates [10]. Moreover, Thompson et al., in a study involving 21,315 participants, reported that supplementing with vitamin D may lower the risk of major cardiovascular events (MACEs) [11]. Given that many individuals may have 25-hydroxyvitamin D [25(OH)D] levels below 30 ng/mL (75 nmol/L), it is reasonable to suggest initiating vitamin D supplementation at 4000 IU per day or an equivalent weekly dose [12,13].

The metabolites 3-epi-25(OH)D₂ and 3-epi-25(OH)D₃ are stereoisomers of 25(OH)D₂ and 25(OH)D₃, respectively. They share the same molecular formula and weight, resulting in similar spectroscopic characteristics and identical fragmentation patterns. However, they differ in the stereochemistry of the hydroxyl group at carbon-3, where the orientation changes from α to β [14,15]. Hence, using procedures that do not separate 25(OH)D from their epimers may result in overestimating metabolite concentrations [16]. Notably, in routine laboratory analysis, the measurement of 3-epi-25(OH)D_2_ and 3-epi-25(OH)D_3_ metabolites is not commonly performed [17,18], as these metabolites are generally present at low concentrations and are not the focus of routine clinical practice [18]. According to Torugsa et al. [19], 3-epi-25(OH)D_3_ could account for up to 32.15% of total serum 25(OH)D, and its variation may be explained by genetic polymorphisms of rs12785878 and rs2282679 [20]. In our previous study, we observed that this percentage could reach up to 50% in cardiac patients [21]. These observations support Strathmann et al.’s [22] findings as they reported that including 3-epi-25(OH)D₃ in estimating 25(OH)D₃ could lead to 3% of adults being incorrectly classified as having sufficient levels. The exact biological activity of C3 epimeric metabolites remains unidentified, and it has been discussed broadly using *in vitro* models; however, limited research articles have been published in the *in vivo* models [20,23,24]. It is worth noting that the epimeric forms have less affinity to the vitamin D receptor, resulting in overall decreased activity compared with 25(OH)D and representing partial inactivation of the hormone; for example, 3-epi-1α,25(OH)_2_D binds with 35–120-fold lower affinity compared with 1α,25(OH)_2_D [25,26]. Several studies reported a positive correlation between C3-epimer and 25(OH)D levels in pregnancy [27], chronic liver disease [28], thyroid disorders [29], and cardiovascular disease [21]. Still, different associations between the epimer and diseases were reported; for instance, Stokes and Vomer [28] did not observe an effect of cirrhosis on epimer concentrations, while Bennett et al. [27] indicated the association of 3-epi-25(OH)D with HbA1c in women with type 1 diabetes.

This study aimed to quantify vitamin D hydroxy metabolites (25(OH)D_2_, 25(OH)D_3_, their epimers 3-epi-25(OH)D_2_ and 3-epi-25(OH)D_3_, and the active form of vitamin D, 1α,25(OH)_2_D_3_) in patients with cardiovascular disease. Additionally, plasma levels of metabolites were determined in samples collected from adult healthy volunteers with no history of circulatory system diseases. As far as we know, no prior studies have been conducted on this topic in the Polish population. This is particularly significant given the scarcity of publications focusing on 3-epi-25(OH)D_2_ in patients with cardiovascular disease. By addressing this gap, our study provides novel insights into the prevalence and clinical relevance of these metabolites in a specific and previously unexamined population.

## Materials and methods

### Study population

We included 35 healthy volunteers and 27 adult patients, including patients with coronary artery disease (*n* = 5) and venous thrombosis (*n* = 22). We recruited patients with cardiovascular disease who were receiving treatment at the Department of Vascular and Endovascular Surgery, Angiology, and Phlebology at Poznan University of Medical Sciences (PUMS). Any patient with vitamin D supplementation, acute myocardial infarction, current liver dysfunction, malignancies, or impaired renal function (defined as a serum creatinine concentration greater than 2 mg/dL) was excluded from the study. Additionally, samples of adult healthy volunteers without a history of cardiovascular disease were collected in the Department of Endocrinology, Metabolism, and Internal Medicine at PUMS. The study was planned and conducted according to the ethical principles of the Declaration of Helsinki [30]. Each subject received information about the study design, objectives, and risk and signed the informed consent form. The Ethical Committee at PUMS approved the study protocols (approval numbers: 873/19, 58/20, 201/21, and 510/21).

The 5 mL blood sample was collected through a single venipuncture into an EDTA tube after an overnight fast. To separate the plasma, the blood samples were centrifuged at 1620xg for 10 minutes, and aliquots were stored at −80°C until analysis by LC-MS/MS and immunoassay. Patients and healthy volunteers were categorized according to their 25(OH)D_3_ levels (sufficient, ≥20 ng/mL; insufficiency, 12–19.99; <12 ng/mL, deficient) [31].

### Determining concentrations of vitamin D hydroxy metabolites

To measure the concentrations of vitamin D metabolites—25(OH)D₂, 25(OH)D₃, 3-epi-25(OH)D₃, and now 3-epi-25(OH)D₂—we modified our previously validated UPLC-MS/MS method [21]. The original method did not account for 3-epi-25(OH)D₂, necessitating this adjustment. In the updated protocol, 200 μL of each patient’s plasma sample was combined with 20 μL of methanol and 20 μL of a deuterated internal standard, D₆-25(OH)D₃. To extract the analytes, we added two separate 1,000 μL portions of hexane to the mixture. After thorough mixing, the hexane layers were collected and evaporated under nitrogen to remove the solvent. The remaining residue was reconstituted in a methanol–water solution to prepare it for analysis. We injected a 10-μL aliquot of this prepared solution into a UPLC Nexera system connected to a triple quadrupole mass spectrometer (LCMS-8030 model by Shimadzu, Kyoto, Japan). Separation of the four targeted metabolites was achieved using a Kinetex 2.6 μm F5 analytical column (dimensions: 100 mm × 2.1 mm) from Phenomenex (Torrance, CA, U.S.A.). The mobile phase consisted of methanol and water in a 70:30 vol ratio, supplemented with 0.1% formic acid to enhance ionization efficiency. For detection, we employed positive electrospray ionization mode in the mass spectrometer, which is effective for ionizing the vitamin D metabolites under study. Our method proved to be both accurate and precise, with a linear response for analyte concentrations ranging from 1 to 100 ng/mL.

To determine the concentration of 1α,25(OH)₂D₃, we used a competitive enzyme-linked immunosorbent assay (ELISA) kit—specifically, the DHVD3 kit from St. John’s Laboratory (London, U.K). The assay was performed according to the manufacturer’s instructions, ensuring reliable and reproducible results.

### Statistical analysis

Statistical analysis was performed using Statistica 13.3 with Plus Kit 3.0 (TIBCO Software Inc., Tulsa, OK, U.S.A.). The Shapiro–Wilk test was applied to evaluate the normality of metabolite distributions. Variables with a normal distribution were expressed as mean ± standard deviation (SD), while non-normal variables were reported as median and interquartile range (IQR). Correlation analysis was conducted using Spearman’s rank correlation coefficient, a nonparametric method. Logistic regression results were expressed as odds ratios (ORs) with corresponding 95% confidence intervals (CIs). Statistical significance was determined at a *P*-value threshold of <0.05 for all tests.

## Results

### Demographic and baseline features

Patients were numerically older, with a mean age of 64.1 years, compared with 36.4 years for healthy volunteers. A greater proportion of patients were male (51.9%) compared with 5.7% in the healthy group, while females accounted for 48.1% of patients and 94.3% of healthy volunteers. Weight, height, and BMI values were comparable between the groups. In terms of vitamin D status, vitamin D insufficiency was prevalent in approximately 52% of the patients compared with 37% of healthy volunteers (Table 1).

**Table 1:** Baseline characteristics of patients and healthy volunteers (values are presented as the number of participants, *n* (%), or mean ± (SD)).

	Patients (*n* = 27)	Healthy volunteers (*n* = 35)
Age (years)	64.1 (14.8)	36.4 (12.3)
Males [*n*, %]	14 (51.9)	2 (5.7)
Females [*n*, %]	13 (48.1)	33 (94.3)
Weight [kg]	78.5 (17.1)	75.4 (14.4)
Height [cm]	169.8 (8.2)	166.4 (6.6)
BMI [kg/m^2^]	27.1 (4.4)	27.3 (5.1)
25(OH)D_3_ ≥ 20 ng/mL (*n*, %)	13 (48.1)	22 (62.9)
25(OH)D_3_ < 20 ng/mL (*n*, %)	14 (51.9)	13 (37.1)
Total 25(OH)D_3_ ≥ 20 ng/mL (*n*, %)	13 (48.1)	25 (71.4)
Total 25(OH)D_3_ < 20 ng/mL (*n*, %)	14 (51.9)	10 (28.6)
Total 25(OH)D_3_ 12–19.99 ng/mL (*n*, %)	6 (22.2)	6 (17.1)
Total 25(OH)D_3_ < 12 ng/mL (*n*, %)	8 (29.6)	4 (11.4)

### Vitamin D metabolites and disease status

Comparable levels of 25(OH)D_3_ and 25(OH)D_2_ were observed for patients and healthy volunteers; however, all patients had 3-epi-25(OH)D_2_ levels below the detection limit (2 ng/mL); hence, it was not possible to determine their levels of % 3-epi-25(OH)D_2_ (Table 2).

**Table 2:** The values of vitamin D metabolites in healthy volunteers and patients are given as median (Q1–Q3).

	Patients	Healthy volunteers
25(OH)D_3_ (ng/mL)	20.4 (10.7–33.7)	25.3 (16.4–34.5)
3-epi-25(OH)D_3_ (ng/mL)	2.2 (2.2–2.5)	3.1 (2.7–4.4)
25(OH)D_2_ (ng/mL)	8.1 (7.8–8.4)	6.7 (4.7–13.4)
3-epi-25(OH)D_2_ (ng/mL)	< LLOQ	4.2 (3.5–6.8)
Total 25(OH)D_3_^α^(ng/mL)	20.4 (12.3–36.2)	25.9 (20–37.8)
% 3-epi-25(OH)D_3_^β^ (%)	20.4 (12.3–36.2)	12 (7.3–18.6)
Total 25(OH)D_2_γ (ng/mL)	8.1 (7.8–8.4)	9.2 (6.2–13.4)
% 3-epi-25(OH)D_2_^δ^ (%)	ND	38.2 (36.4–41.1)
1α,25(OH)_2_D_3_ [pg/mL]	44.6 (35.7–128.3)	67.9 (35.9–138.0)

α – 25(OH)D_3_+3-epi-25(OH)D_3_

β – 3-epi-25(OH)D_3_ / (25(OH)D_3_+3-epi-25(OH)D_3_)

γ – 25(OH)D_2_+3-epi-25(OH)D_2_

δ – 3-epi-25(OH)D_2_ / (25(OH)D_2_+3-epi-25(OH)D_2_)

LLOQ , lower limit of quantification. ND , not determined.

### Vitamin D metabolites and demographic characteristics

For healthy volunteers, the levels of 25(OH)D_2_ and % 3-epi-25(OH)D_3_ were negatively correlated with weight (*R* = −0.429, *P* = 0.046) and (*R* = −0.523, *P* = 0.031), respectively (Table 3). No significant correlation was observed between the demographic characteristics and patients with cardiovascular disease (Table 3).

**Table 3:** Correlations between vitamin D metabolites and demographic characteristics.

	Age	Weight	BMI	Height
**Healthy volunteers**				
25(OH)D_3_	−0.125	0.218	0.047	0.171
3-epi-25(OH)D_3_	0.128	−0.357	−0.462	−0.207
25(OH)D_2_	−0.413	−0.429^α^	−0.174	−0.389
3-epi-25(OH)D_2_	−0.396	0.079	−0.195	0.297
Total 25(OH)D_3_	−0.158	0.161	0.020	0.124
% 3-epi-25(OH)D_3_	0.041	−0.523^α^	−0.451	−0.381
Total 25(OH)D_2_	−0.415	−0.393	−0.269	−0.354
% 3-epi-25(OH)D_2_	0.372	0.212	0.036	0.127
1α,25(OH)_2_D_3_	−0.359	−0.143	0.338	−0.311
**Patients with cardiovascular disease**				
25(OH)D_3_	−0.190	−0.196	−0.138	−0.152
3-epi-25(OH)D_3_	−0.462	NA	0.500	NA
25(OH)D_2_	−0.580	NA	NA	NA
Total 25(OH)D_3_	−0.179	−0.196	−0.138	−0.152
% 3-epi-25(OH)D_3_	0.300	NA	0.500	NA
Total 25(OH)D_2_	−0.580	NA	NA	NA
1α,25(OH)_2_D_3_	−0.319	−0.059	−0.155	0.224

α –Marked correlations are significant at *P*<0.05.

NA , not applicable.

### Relationship between vitamin D metabolites and their epimers

**Figure 1:**
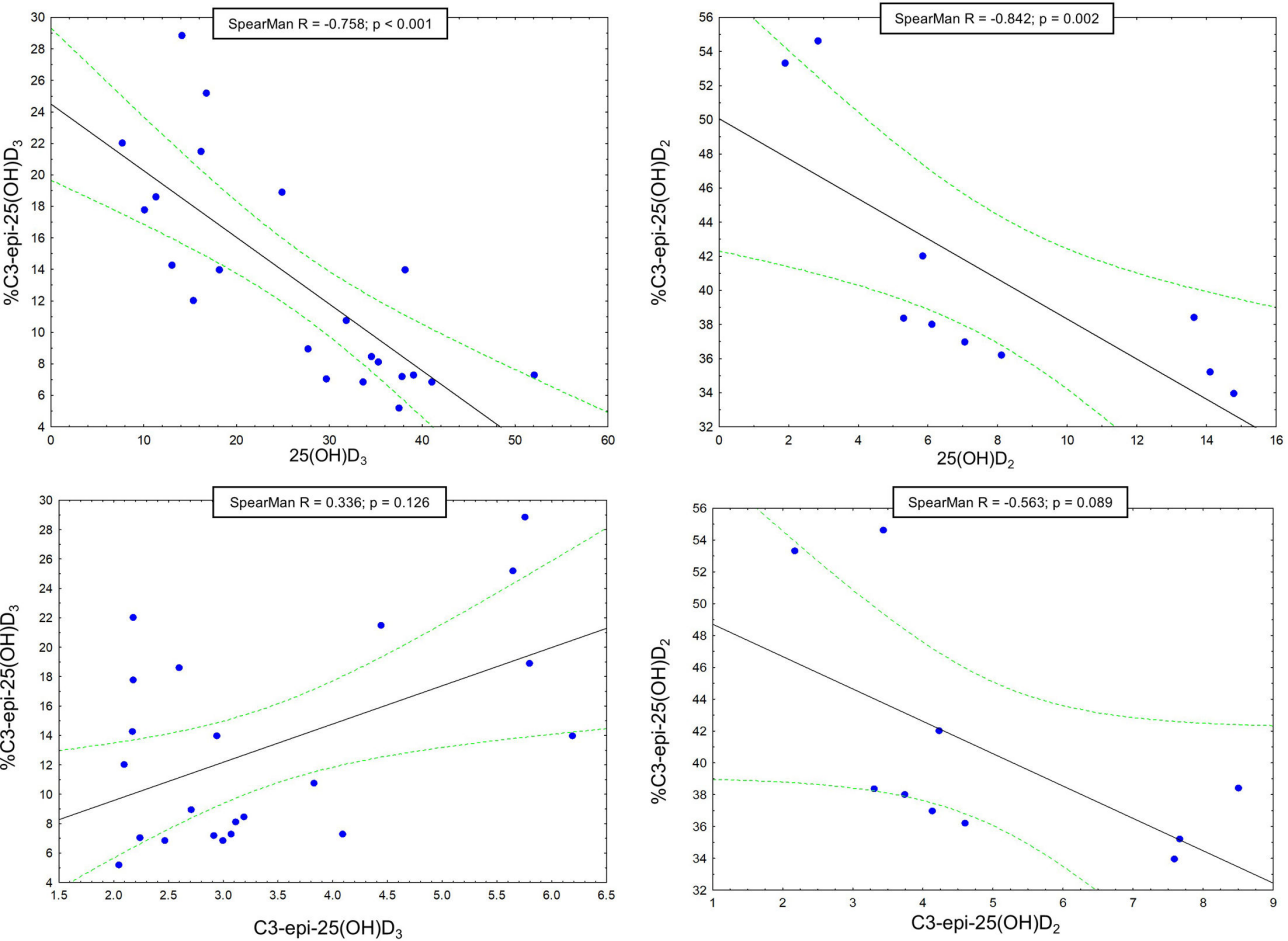
Correlations between vitamin D metabolites and their epimers.

Overall, a significant negative correlation was found between 25(OH)D_3_ and % 3-epi-25(OH)D_3_ (*R* = −0.758, *P* < 0.001), as well as between 3-epi-25(OH)D_2_ and % 3-epi-25(OH)D_2_ (*R* = −0.842, *P* = 0.002). However, no significant correlation was detected between 3-epi-25(OH)D_3_ and % 3-epi-25(OH)D_3_ or between 3-epi-25(OH)D2 and % 3-epi-25(OH)D_2_ (Figure 1). Similarly, no significant correlation was observed between 1α,25(OH)_2_D_3_ and 25(OH)D_3_, 25(OH)D_2_, or their respective percentages.

### Cardiovascular disease predictors

The univariate analysis identified two significant predictors for cardiovascular disease. Age was found to be a significant factor, with an OR of 1.12 (95% CI: 1.07–1.18, *P* < 0.001), indicating that the risk of cardiovascular disease increases with advancing age. Male sex was another significant predictor, with an OR of 17.77 (95% CI: 3.54–89.31, *P* < 0.001), suggesting that males have a substantially higher likelihood of developing cardiovascular disease compared with females (Table 4). We also performed a backward stepwise multiple regression analysis including all the variables listed in the table. However, none of the variables remained significant in the final model. When the analysis was limited to demographic characteristics, only age and sex persisted as significant predictors.

**Table 4:** Univariate predictors for patients with cardiovascular disease.

	*b* coeff.	*b* error	Wald stat.	*P*	Odds ratio	−95% **CI**	+ 95% CI
**Demographic characteristics[Table-fn T4_FN1]**							
Age	0.114	0.025	20.775	0.000	1.121	1.067	1.178
Sex (male)	2.877	0.824	12.200	0.000	17.769	3.535	89.311
Weight	0.014	0.018	0.561	0.454	1.014	0.978	1.050
Height	0.067	0.041	2.718	0.099	1.070	0.987	1.159
BMI	−0.011	0.058	0.038	0.845	0.989	0.882	1.109
25(OH)D_3_≥20 ng/mL	−0.942	0.528	2.847	0.09	0.39	0.131	1.164
**Vitamin D metabolites**							
25(OH)D_3_	−0.005	0.018	0.078	0.780	0.995	0.960	1.031
3-epi-25(OH)D_3_	−2.030	1.301	2.435	0.119	0.131	0.010	1.682
25(OH)D_2_	−0.033	0.117	0.080	0.778	0.968	0.770	1.216
Total 25(OH)D_3_	−0.011	0.018	0.386	0.535	0.989	0.954	1.024
% 3-epi-25(OH)D_3_	−0.036	0.081	0.195	0.659	0.965	0.823	1.131
Total 25(OH)D_2_	−0.117	0.110	1.123	0.289	0.890	0.717	1.104
1α,25(OH)_2_D_3_	−0.001	0.003	0.143	0.706	0.999	0.994	1.004

† A backward stepwise multiple regression analysis of the demographic characteristics identified age (OR = 1.11, 95% CI: 1.049–1.174, *P* < 0.001) and sex (OR = 19.893, 95% CI: 1.121–326.566, *P* = 0.036) as significant predictors.

## Discussion

We found that vitamin D insufficiency (<20 ng/mL) was observed in approximately 52% of the patients and 29% of healthy volunteers. However, when the classification was based on a total 25(OH)D_3_, 37% of healthy volunteers showed vitamin D insufficiency—hence, approximately 9% were misclassified when epimers were not separated. While similar levels of 25(OH)D_3_ and 25(OH)D_2_ were seen in both groups, the observed levels of the epimeric form 3-epi-25(OH)D_3_ appeared approximately 1.7 times higher in healthy volunteers, and 3-epi-25(OH)D_2_ was undetectable in patients. However, only higher age and being male were univariate predictors for patients with cardiovascular disease. Also, we observed a negative correlation between 25(OH)D_2_ and 25(OH)D_3_ and the percentage of their epimeric forms.

Vitamin D’s association with cardiovascular disease remains an ongoing debate. The D-Health Trial [11] was a large-scale, double-blind, randomized controlled trial (RCT) conducted in Australia to assess the effects of vitamin D supplementation on the incidence of MACE. Participants were randomly assigned to receive either a monthly dose of 60,000 IU of vitamin D_3_ or a placebo, with the intervention lasting up to five years [11]. In their analysis of the D-Health Trial, Thompson et al. reported that monthly supplementation with 60,000 IU of vitamin D3 for up to five years was associated with a reduced incidence of MACE, particularly for the myocardial infarction and coronary revascularization, despite the fact that the null was included in the CI for the hazard ratio (HR) of the latter (HR 0.89, 95% CI: 0.78–1.01) [11]. When comparing the D-Health study to other large RCTs, VITAL (daily supplementation with vitamin D3 (2000 IU) or omega-3 fatty acids (1 gm)) [32] and ViDA (monthly dose of 100,000 IU of vitamin D_3_ or a placebo) [33], Thompson et al. noted that VITAL did not demonstrate a protective effect against MACE, including myocardial infarction and coronary revascularization. Likewise, the ViDA study found no protective effect of vitamin D supplementation against the total cardiovascular disease (HR 1.02, 95% CI: 0.87–1.20) or stroke (0.95, 95% CI: 0.55–1.62).

However, in the VITAL study, as highlighted above, the dosing regimen was daily, unlike the ViDA and D-Health Trials, where the monthly dose was administered. Evidence suggests that daily dosing may be more beneficial for specific health outcomes, such as reducing cancer mortality and combating infections. With this mentioned, it is important also to highlight that the monthly dosing schedule used in D-Health might have contributed to better adherence compared with VITAL. For instance, in the D-Health Trial, 80% of participants reported adhering to approximately 80% of the prescribed study tablets, but in the VITAL Trial, around 80% of participants reported consuming only approximately two-thirds of the study tablets [32].

In our study, the levels of 25(OH)D_3_ were comparable between healthy volunteers and patients. We observed that the levels of the epimeric form 3-epi-25(OH)D_3_ appeared approximately 1.7 times higher in healthy volunteers. Furthermore, levels of 3-epi-25(OH)D_2_ were all below the LLOQ for patients with cardiovascular disease (<2 ng/mL). In contrast, at least 50% of healthy volunteers had 3-epi-25(OH)D_2_ levels within the range of 3.5–7.6 ng/mL. This information may suggest a substantial decline of the 3-epi-25(OH)D_2_ epimer in patients with cardiovascular diseases. However, such an interpretation should be further confirmed with a more accurate analysis method.

Al-Zohily et al. [20] highlighted the possibility of epimerization at the C3 position for all major vitamin D metabolites. However, only a limited number of laboratories globally account for the potential inaccuracies in measurements caused by the overlap of C3-epimers. Moreover, when measuring vitamin D levels in the blood of healthy adults using mass MS/MS, there is little-to-no significant impact from C3-epimer interference due to the high accuracy and sensitivity of the method. We have observed this in our study as the levels of 3-epi-25(OH)D_3_ appeared approximately 1.7 times higher in healthy volunteers, accounting for 9% of them to be misclassified according to vitamin D status. In other words, when we measured total 25(OH)D_3_, 25 out of 35 healthy volunteers were found to have sufficient vitamin D levels. However, after separating the epimer and calculating vitamin D status based solely on 25(OH)D_3_, 22 out of 35 healthy volunteers were identified as having sufficient vitamin D levels.

Notably, studies on genetic models have shown that the factors influencing the formation and regulation of C3-epimers are distinct from those that affect non-C3-epimers; for instance, Torugsa et al. [19] showed that the minor G allele showed a reduction in the levels of both non-C3-epimers and C3-epimers, but minor T allele demonstrated a tendency for higher levels of C3-epimers only. This aligns with findings from our previous study [34], where the levels of 3-epi-25(OH)D_3_ were shown to vary based on the type of VDR polymorphism.

Research on the epimeric forms of vitamin D in cardiovascular disease remains limited, particularly for 3-epi-25(OH)D_2_, although growing evidence highlights the significance of 3-epi-25(OH)D_3_ biomarkers. Arroyo et al. [35] suggested that variations in 3-epi-25(OH)D_3_ levels might influence cardiovascular functional capacity in individuals with advanced chronic kidney disease (CKD). In their study of 165 patients with advanced CKD from the Cardiopulmonary Exercise Testing in Renal Failure and After Kidney Transplantation (CAPER) cohort, they found that patients with low serum 3-epi-25(OH)D_3_ levels (<0.4 ng/mL) exhibited significantly reduced peak oxygen consumption (VO_2_Peak), a measure of peak aerobic capacity, compared with those with higher levels (≥0.8 ng/mL) [35]. VO_2_Peak, recognized as the gold standard for assessing cardiovascular fitness, is a strong prognostic marker in chronic heart failure [36] and is generally associated with improved health and longevity in the broader population [37]. The lower VO_2_Peak values reported by Arroyo et al. [35] suggest that 3-epi-25(OH)D_3_ could play a regulatory role in cardiovascular functional capacity in patients with advanced CKD.

Chen et al. [38], in a cross-sectional case–control study involving 3086 healthy participants and 4,120 patients, identified the percentage of C3-epi-25(OH)D_3_, rather than its absolute level, as a potential biomarker for pathological changes in multiple conditions. They found that an elevated %C3-epi-25(OH)D_3_ was associated with increased odds of hypovitaminosis D (OR 1.23, 95% CI: 1.11–1.36, *P* < 0.001), coronary artery disease (OR 1.33, 95% CI: 1.17–1.52, *P* < 0.001), stroke (OR 1.28, 95% CI: 1.17–1.48, *P* < 0.001), and thrombosis (OR 1.20, 95% CI: 1.08–1.34, *P* < 0.001). Interestingly, adjusting for 25(OH)D_3_ levels significantly diminished the predictive strength of %C3-epi-25(OH)D_3_ for these diseases. However, the association remained statistically significant for thrombosis, with an adjusted OR of 1.23 (95% CI: 1.05–1.44, *P* = 0.009), highlighting its potential as a specific risk indicator for this condition.

### Limitations

Our study is the first to report the determination of a group of five vitamin D hydroxy metabolites, 25(OH)D_2_, 25(OH)D_3_, 3-epi-25(OH)D_3_, 3-epi-25(OH)D_2_, and 1α,25(OH)D3 in Polish patients with cardiovascular diseases and healthy volunteers. However, we must still clarify the limitations of this study. First, due to the higher number of women in the group of healthy volunteers and the age gap, we did not perform a statistical comparison between the healthy volunteers and patients with cardiovascular disease. Therefore, we used a univariate model, which confirmed age as a predictor of the disease, but none of the vitamin D hydroxy metabolites were identified as predictors. Second, the role of seasonal analysis was ignored in our study, which might have influenced the levels of the metabolites, as we elaborated in our previous observational study [39]. Third, we used univariate as predictors, but backward stepwise regression did not show significant results. Therefore, the result of this study should not be generalized until a larger sample size confirms it. Fourth, not all parameters were detected for all participants. Fifth, we lack data on the patient’s behaviors and lifestyle, which may impact vitamin D body status.

## Conclusion

Overall, only 56.5% of the participants had sufficient vitamin D levels, underscoring the potential need for vitamin D supplementation in this population. Patients with cardiovascular disease demonstrated numerically lower levels of vitamin D epimeric metabolites and calcitriol. However, neither vitamin D levels nor its epimeric metabolites, or calcitriol were identified as predictors of cardiovascular disease.

The observed association between vitamin D hydroxy metabolites and their epimeric forms highlights the need for further research involving larger and more diverse populations to better elucidate the relationship between vitamin D metabolites and cardiovascular health.

## Data Availability

The data presented in this study are available on request from the corresponding author.

## Abbreviations

CIs: confidence intervals
CKD: chronic kidney disease
ELISA: enzyme-linked immunosorbent assay
HR: hazard ratio
IQR: interquartile range
LLOQ: lower limit of quantification
MACE: major cardiovascular events
ORs: odds ratios
PUMS: Poznan University of Medical Sciences
RCT: randomized controlled trial
RCT: randomized controlled trial
SD: standard deviation
UPLC-MS/MS: ultra-performance liquid chromatography-mass spectrometry/mass spectrometry
